# Family Accommodation Scale for Sensory Over-Responsivity: A Measure Development Study

**DOI:** 10.3389/fpsyg.2022.867508

**Published:** 2022-05-16

**Authors:** Ayelet Ben-Sasson, Tamar Yonit Podoly, Eli Lebowitz

**Affiliations:** ^1^Department of Occupational Therapy, Faculty of Social Welfare and Health Sciences, University of Haifa, Haifa, Israel; ^2^Association for Children at Risk, Kfar Saba, Israel; ^3^Department of Psychology, Bar-Ilan University, Ramat Gan, Israel; ^4^Child Study Center, Yale University School of Medicine, New Haven, CT, United States

**Keywords:** sensory over-responsivity, family accommodations, children, measure development, sensory modulation

## Abstract

Family accommodation refers to the attempt of family members (most often parents) to prevent their child’s distress related to psychopathology. Family accommodation can limit meaningful participation in personal and social routines and activities. Accommodation has been studied extensively in the context of childhood anxiety and has been linked to greater impairment, and poor intervention outcomes. Like anxiety, sensory over-responsivity (SOR) symptoms are associated with heightened distress and thus, may also be accommodated by family members. The current study describes the validation of a new pediatric family accommodation scale for SOR. Parents of 301 children ages 3–13 years completed an online survey, of which 48 had medical or developmental conditions. The survey included the Child Sensory Profile 2 and the newly developed family accommodation scale for sensory over-responsivity (FASENS). Three Sensory Profile 2 scores were analyzed: SOR, sensory under-responsivity and sensory seeking. The FASENS consists of 18 items; 12 describing the frequency of accommodation behaviors and 6 describing the impact of the accommodation on the wellbeing of the family and the child. Results indicated that the FASENS has high internal consistency (α = 0.94) as well as a significant 3-factor confirmatory model fit: (1) accommodations (i.e., avoidance and changes), (2) family impact, and (3) child impact. FASENS scores significantly correlated with SOR symptoms (*r* = 0.52–0.60, *p* < 0.001). However, they also correlated with under-responsivity and seeking (*r* = 0.33–0.42, *p* < 0.001). Parents of children with health conditions reported significantly higher FASENS scores (*p* < 0.002), which corresponded with their child’s significantly higher sensory scores (*p* < 0.001). Family accommodations for SOR occur to some extent in the general population, but their prevalence and impact are significantly greater when the child has a health condition, in addition to SOR. Additional research is needed to explore whether these accommodations are adaptive and whether families and children would benefit from learning to reduce them, as with anxiety.

## Introduction

The development of sensory modulation, the ability to execute an adapted behavior in response to the sensory environment, is a complex process which relates to several factors. These include developmental age, temperament, innate regulation ability, and the degree of previous exposure to stimuli (Williamson and Anzalone, 2001). Typically developing children differ in their level of sensory over-responsivity (SOR) and in the degree to which it interferes with their participation in daily activities (Bar-Shalita et al., 2008; Ben-Sasson et al., 2010). Despite the weight of personal characteristics in the ability to regulate and adapt to the sensory environment, the importance of the family environment and reactions should not be underestimated. Families define opportunities for sensory exposure, as well as affective modeling of coping in stressful situations. Families vary in the way they respond to their child’s sensory sensitivities. The current study aimed to develop a tool for quantifying how families accommodate to their child’s sensory sensitivities, and the distress caused to the child and/or family because of these interactions.

Family accommodation describes attempts of family members to reduce their child’s distress by avoiding the source of fear, taking part in rituals, reassuring, and changing their routines and activities. It is important to identify family accommodations as while they may provide immediate relief, they predict greater symptom severity (i.e., higher anxiety levels, more rituals, and compulsions), lower levels of functioning (more avoidant behaviors), and poor intervention outcomes (Storch et al., 2007; Peris et al., 2008; Lebowitz et al., 2012, 2013; Strauss et al., 2015; Feldman et al., 2019; Shimshoni et al., 2019). Family accommodations have primarily been studied among children with OCD and anxiety disorders (Storch et al., 2007; Lebowitz et al., 2013) and to some degree in ASD (Feldman et al., 2019). Research shows that although the types of accommodations among different fear-based disorders may differ, the frequency of accommodations remains the same. These conditions share repetitive, catastrophic thoughts, experiences of fear, avoidance, and seeking a secure state (Reuman and Abramowitz, 2018). Since SOR is associated with heightened anxiety (Ben-Sasson et al., 2009; Conelea et al., 2014), we hypothesized that family members would accommodate some sensory symptoms, even when it comes to families with typically developing children.

Although family accommodations reflect the parents’ intention to reduce their child’s distress, paradoxically these strategies tend to reinforce the child’s distress and avoidance and inhibit the child’s ability to self-regulate (Norman et al., 2015). Many parents report distress when performing accommodations, while when they do not accommodate their child’s anger, distress and worry increases (Reuman and Abramowitz, 2018). Some children cannot complete certain tasks without accommodations, which pressures parents to construct them (Lebowitz et al., 2014). Therefore, understanding the types of family accommodations and their effects on the child and parents is important for understanding the delicate child-family dynamics surrounding a disorder and for facilitating healthier child-family interactions.

Sensory modulation reflects the individual’s ability to respond adaptively to interoceptive and exteroceptive stimuli. This ability reflects continuous information processing of the intensity, duration and frequency of stimulation enabling attention to relevant stimuli while filtering out background stimuli (Brown et al., 2018). Sensory modulation also involves maintaining an arousal level adjusted to the environment and activity (Hong, 2015). Sensory modulation disorder (SMD) is diagnosed when a difficulty in the process of sensory modulation impairs daily functioning. According to Miller et al. (2007), there are three types of SMD: SOR which is an intense and over-sensitive response to mundane stimulation; Sensory Under-responsivity, a lack of responsiveness and inattention to every day sensory stimulation; or Sensory Seeking, a constant sensory search and craving. Sensory modulation traits follow the same classification. The current study focuses on the design of a tool for quantifying the family’s response to the child’s SOR symptoms by studying its distribution in a non-clinical sample.

Children with elevated SOR experience many everyday stimuli at home and in the community as bothersome, unbearable, and overwhelming. This is manifested in behavioral avoidance, elevated distress, anxiety, and/or active resistance of the sensory exposure. Consequently, those with SOR find it difficult to participate in some activities and feel anxious before and during the encounter with stimuli (Parham and Mailloux, 2005). Evidence shows that a child’s SOR is associated with limited participation in leisure activities and requires changes and restrictions in family activities and routines (DeGrace, 2004; Bar-Shalita et al., 2008; Epstein et al., 2008; Bagby et al., 2012). Studies dealing with the implications of SOR on quality of life and family well-being demonstrate the challenges and stress associated with having a child with SOR. Parents of these children report increased restrictions in their personal and social activities (Carter et al., 2011), they experience more burden and challenges, especially when the mothers have sensory difficulties of their own (Turner et al., 2012; Gafni-Lachter et al., 2021).

The increased irritability and distress associated with SOR can lead some parents to try to minimize their child’s distress. To meet the child’s sensory needs, parents build strategies and routines which enable participation in activities within the home (Spagnola and Fiese, 2007). These efforts include changing schedules and finding resources to meet their child’s needs, which can disrupt family cohesion (Spagnola and Fiese, 2007). Parents of children with ASD, described the difficulty that arises from trying to balance responding to the child’s sensory difficulties, while maintaining flexibility in daily routines (Schaaf et al., 2011). The family’s restrictions, adjustments, and adaptations around the child’s SOR, help reduce the child’s exposure to the bothersome sensations and avoid outbursts. Changes in family life due to SOR have not been evaluated from a family accommodation perspective and it is not known whether family accommodations maintain or exacerbate sensory avoidance. Developing a tool to characterize family accommodations for SOR is a first step in enabling such an assessment.

Existing family accommodation scales originated from tools developed for adults with OCD (Calvocoressi et al., 1999). Later, scales were developed for children with OCD (Peris et al., 2008), anxiety disorders (Lebowitz et al., 2013; Benito et al., 2015) and ASD (Feldman et al., 2019). These scales share the assessment of (1) the frequency of accommodations (e.g., enabling child’s avoidance of feared situation). And (2) severity of consequences of not providing the accommodations for child and family wellbeing (e.g., level of distress when accommodation is not delivered). Therefore, the sensory family accommodation tool designed quantified these two aspects.

To summarize, the literature reviewed indicates that having SOR is a cause of child anxiety and avoidance as well as family distress; thus, we predict that it is likely to evoke accommodations. Identifying the specific accommodations associated with sensory symptoms can reveal precipitating, perpetuating, and protective environmental factors. The current research sought to establish the reliability and validity of a new tool, the Family Accommodations Scale for Sensory Over-Responsivity (FASENS), by investigating the:

(1) internal reliability and structure validity of the tool,
(2) frequency and impact of sensory-related family accommodations in the general population,
(3) discrimination of the FASENS scores between children with and without health conditions associated with elevated SOR,
(4) convergent validity of the FASENS scores with the child’s sensory profile scores.

## Materials and Methods

### Procedure

The study was approved by the Ethics Committee of University of Haifa. Parents were recruited through social media and other social networks by the research team and by undergraduate students in a research course at University of Haifa. Using a link, parents entered a Qualtrics survey in which they signed consent to participate in the study and completed the questionnaires for up to 30 min. If a family had more than one qualifying child, parents were asked to report on one child only.

### Participants

Included were children ages of 3–13 years living in a two-parent household, to avoid a potential effect of single parenting on family interactions (Chapple, 2009). Parents were proficient in Hebrew. A total of 301 parents completed the survey intended for the general population. Children were an average of 8.2 years old (SD = 2.7) and 161 (53.5%) were males. See Table 1 for background information. Of the 301 parents, 48 (15.95%) reported a significant medical or developmental condition. This subgroup will be referred to from here on as the “Conditions” group. Excluded from this group were children with corrected issues such as vision, chronic ear infections, or who attended a few sessions of therapy in the past.

**TABLE 1:** Background characteristics.

Variable	Result
Position in family N (%)^a b^	
Only child	16 (5.40%)
First of several^b^	118 (40.0%)
Middle^b^	75 (25.40%)
Last^b^	86 (29.20%)
Mother’s age, M (SD)	38.28 (5.67)
Father’s age, M (SD)	41.14 (5.82)
Mother’s years of education, M (SD)	16.21 (3.70)
Father’s years of education, M (SD)	15.40 (3.96)
Mother full time employment, N (%)^a^	173 (57.50%)
Father full time employment, N (%)^a^	248 (82.40%)
Developmental and medical issues N (%)*	48 (15.95%)
ADHD	15
Allergies (e.g., skin, food)	8
Growth (e.g., FTT, Obesity)	5
Developmental coordination disorder	2
Sensory modulation problems	6
Mental health difficulties	6
Developmental delays (e.g., general, language)	7
Chronic medical condition (e.g., epilepsy, heart condition)	6
Pervasive developmental condition (e.g., ASD)	2

*Note: ADHD, attention deficit hyperactivity disorder; FTT, failure to thrive; and ASD, autism spectrum disorder.*

*The categories are not mutually exclusive.

*^a^These variables were missing for some of the sample.*

*^b^Of 2–7 children, with 73.4% 2–3 children in family.*

### Measures

#### Family Accommodations Scale for Sensory Over-Responsivity

The FASENS^1^ was designed as a caregiver questionnaire to assess family accommodations related to children’s SOR symptoms. The questionnaire starts with explaining SOR symptoms, listing examples of behaviors in auditory, visual, tactile, movement, smell and taste modalities. Next, are 18 items to rate relative to these symptoms (see Supplementary Table 1). Twelve items describing family accommodation behaviors of avoidance and changes implemented by family members in the past month, on a 5-point Likert scale from 1 “Never” to 5 “Daily.” Four items describe the severity of impact of the accommodation on the child’s function and well-being and 2 describe the impact on the family’s well-being on a 5-point Likert scale from 1 “None” to 5 “Extremely.” Separate mean scores were computed for accommodation frequency, for child impact, and for family impact.

The content of the scale was developed based on the Family Accommodation Scale Anxiety (FASA) in terms of item phrasing, domains, and Likert scales. FASENS items were designed to reflect family challenges related to SOR as reported in the literature and based on the clinical expertise of the first two authors. The content validity of the first draft of the questionnaire was tested. Three clinical pediatric experts and three parents of elementary school-age children were asked to review the measure for the degree to which items measure family accommodations, clarity, and missing items. Based on this feedback, the authors revised the scale. The final measure was used in this study.

#### Sensory Profile 2

This caregiver questionnaire evaluates a child’s pattern of sensory processing across six modalities (e.g., auditory, visual, tactile, taste/smell, vestibular, and proprioceptive) involved in daily life activities (Dunn, 2014). Parents rate 86 items on a 5-point Likert scale from 1 “Almost never” to 5 “Almost Always.” The sensory profile items are classified and scored into four quadrant summary scores: Sensitivity, Avoidance, Seeking and Registration (termed under-responsivity in this paper), as well as a SOR composite which is a sum of the Sensitivity and Avoidance scores. The analysis of this study focused on SOR given the high correlation between Avoidance and Sensitivity scores (*r* = 0.81), a method supported by previous research (e.g., Ben-Sasson et al., 2009; Little et al., 2017).

This questionnaire has good internal consistency, 0.71–0.90. Test-retest reliability ranged from 0.83 to 0.97. Inter-rater reliability was 0.70–0.80. Content validity was established through a panel of experts of occupational therapists with expertise in sensory processing. Convergent validity was high between the Sensory Profile 2 and previous Sensory Profile version (Little et al., 2017; Brown et al., 2019). The Hebrew version has been validated and showed strong psychometric properties (Dunn, 2014). This tool has been used to characterize sensory modulation traits in several general population studies (Kientz and Dunn, 1997; Ermer and Dunn, 1998).

#### Demographic Questionnaire

This questionnaire asked for background information, such as child and parents’ ages, gender, birth order in family, parents’ level of education, and child’s medical or developmental status.

### Data Analysis

Internal consistency was tested with Cronbach Alpha. AMOS 27 was used to conduct confirmatory factor analysis of the FASENS items. Three FASENS mean scores were derived: (1) accommodations, (2) family impact, and (3) child impact. Kolmogorov-Smirnov tests of normality indicated that all FASENS mean scores were not normally distributed (*p* > 0.05). Hence, non-parametric tests were applied for testing correlations and group comparisons related to these scores. Discriminant validity of the scale was examined by comparing FASENS scores between typical and conditions groups. FASENS items were compared between groups using Mann–Whitney tests. The associations between FASENS scores and background variables were tested using Spearman correlations for continuous variables, and Mann–Whitney *U* or Kruskal–Wallis tests for comparing 2- or 3-category variables.

## Results

### Internal Consistency

The FASENS items showed a high internal consistency (α = 0.94), with none of the items reducing reliability. Item descriptives are presented in Supplementary Table 1.

### Factor Analysis

**FIGURE 1:**
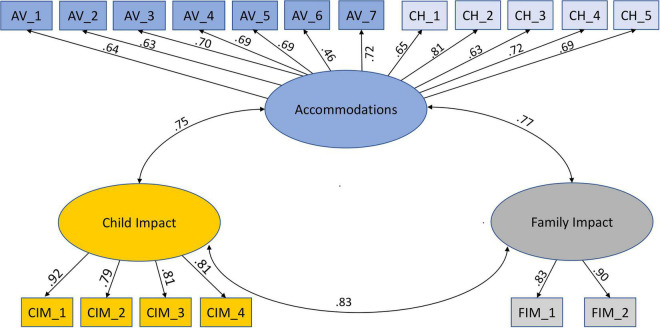
Confirmatory factor analysis model.

Confirmatory factor analysis was conducted in AMOS 27 (see model Figure 1). The very high correlation between FASENS avoidance and changes scores (*r* = 0.79, *p* < 0.001) led us to the analysis of a 3-factor solution with 12 accommodation items in factor 1, 4 child impact items in factor 2 and 2 family impact items in factor 3. The shared variance between four item estimates was accounted for in the model. The model fit was high, as indicated by the ratio between chi and *p*-values = 2.91 under the threshold of 3, CFI = 0.929, IFI = 0.930, and RMSEA = 0.08. All standardized estimates were significant (*p* < 0.001).

### Discriminative Validity

Table 2 presents FASENS and Sensory Profile scores for the total sample and by group. Mann–Whitney *U* tests showed that FASENS scores for parents of typically developing children were significantly lower than for parents of children in the conditions group (see Table 2). This corresponded with MANOVA results indicating the significantly higher Sensory Profile scores, Wilk’s Lambda = 0.90, *F*(3,297) = 11.19, *p* < 0.001, and η^2^ = 0.10 (see Table 2). Mann–Whitney *U* tests comparing FASENS items between typical and conditions groups showed significantly higher scores in five items (*p* < 0.003; see Supplementary Table 1).

**TABLE 2:** Sensory profile and FASENS scores with group comparisons.

	Mean, median (SD), Min-Max			
	Total sample *N* = 301	Typical *N* = 253	Conditions *N* = 48	Statistics*
**FASENS**				
Accommodations	0.62, 0.42 (0.71), 0–3.83	0.56, 0.42 (0.68), 0–3.83	0.90, 0.75 (0.79), 0–3.17	*U* = 4339, *p* = 0.002
Family impact	0.45, 0 (0.74), 0–4	0.38, 0 (0.69), 0–4	0.79, 0.75 (0.86), 0–3	*U* = 4354.50, *p* < 0.001
Child impact	0.72, 0.25 (0.88), 0–4	0.65, 0.25 (0.86), 0–4	1.06, 0.88 (0.91), 0–3	*U* = 4333.50, *p* = 0.001
**Sensory profile**				
SOR	74.48, 72 (28.61), 0–163	70.53, 69 (26.76), 0–159	95.29, 92 (29.3), 26–163	*F*(1) = 3.49, *p* < 0.001, η^2^ = 0.10
Seeking	34.17, 33 (15.115), 0–88	32.59, 31 (14.22), 0–81	42.52, 42.5 (17.02), 5–88	*F*(1) = 18.43, *p* < 0.001, η^2^ = 0.06
Under-responsivity	33.2, 31 (15.58), 0–92	31.59, 31 (15.1) 0–92	41.69, 41 (15.51), 7–81	*F*(1) = 17.88, *p* < 0.001, η^2^ = 0.06

Note: * Mann–Whitney U test was applied for comparisons of FASENS scores and MANOVA for comparisons Sensory Profile patterns scores.

### Convergent Validity

All three FASENS scores were significantly and moderately correlated with Sensory Profile SOR, Seeking, and Under-responsivity scores (see Table 3).

**TABLE 3:** Spearman rho correlations between FASENS scores and sensory profile scores.

	Sensory profile		
	SOR	Seeking	Under-responsivity
**FASENS**			
Accommodations	0.53**	0.42**	0.38**
Child impact	0.60**	0.39**	0.39**
Family impact	0.52**	0.33**	0.42**

Note: **p < 0.001.

### Background Correlates of FASENS

Spearman correlations indicated that child age was mildly negatively correlated with accommodations and child impact scores (*r* = –0.19, *p* = 0.001, *r* = –0.13, *p* = 0.03, respectively). In other words, parents of younger children had higher accommodative behaviors and reported higher child impact score when these accommodations were prevented. Mother’s age was mildly negatively correlated with all three scores (*r* = –0.23, *p* < 0.001, *r* = –0.13, *p* = 0.03, *r* = –0.16, *p* = 0.008, respectively). That is younger mothers tended to report more accommodations, higher family impact and child impact. Father’s age correlated with accommodations and child impact (*r* = –0.19, *p* = 0.001, *r* = –0.14, *p* = 0.02, respectively).

## Discussion

The idea that a child’s living environment can be changed or reorganized with the help of the family in a way that either maintains difficulties or encourages functioning is reflected both in research and clinically (Salloum et al., 2018). Sensory modulation difficulties and specifically SOR, may provoke family accommodations in the same way that anxiety disorders do. Over-responsivity to certain stimuli, can cause the child to avoid these stimuli, and to express severe distress. In response, the parents can accommodate the avoidance by reducing the exposure to the distressing stimulus and by that increasing the avoidance patterns leading to further restriction of child and family meaningful participation. The current study validated a tool designed for assessing family accommodation in the context of SOR.

The FASENS had excellent psychometric properties: it showed high internal consistency and good convergent validity with the Sensory Profile 2 scores. Family accommodation, as measured by the FASENS accommodation score, was significantly correlated with the severity of all sensory patterns, as measured by Sensory Profile 2. Nonetheless, the relation with SOR was the strongest. The relation between accommodation and children’s severity of the psychopathology (i.e., anxiety and OCD) which the accommodation relates to was also shown with previous family accommodation scales (Lebowitz et al., 2013; Flessner et al., 2017). When scrutinizing the FASENS correlations in our study, the magnitude of difference between groups was greatest for the SOR pattern. Given the association with all three sensory profile scores, it should be noted that families are accommodating children who are dysregulated. Since the tool primarily asks parents to think of their child’s sensitivities and highlights the SOR pattern, it is not possible to determine how much of the accommodation is associated with each sensory pattern. As previously mentioned, SOR symptoms can cause children significant distress and anxiety (Carpenter et al., 2019). The child’s anxiety may cause distress and lead to accommodation behaviors of the family, so it is no wonder that the strongest correlation we found was between the SOR pattern and FASENS scores.

Factor analysis confirmed that the FASENS comprises 3 factors: frequency of accommodations, child impact, and family impact. Previous family accommodation scales differed in their number of factors and subscales loaded. The FAS factors were Avoidance of Triggers and Involvement in Compulsions (Flessner et al., 2011) and the FASA factors were Participation and Modification (Lebowitz et al., 2013). Our results indicated that avoidance/participation versus modification/changes items are distributed on one factor rather than two. Overall, the current results underscore the need to evaluate the presence of accommodations separately from their impact on child and family well-being and participation. Replicating this factor analysis in a clinical sample with higher and variable scores is warranted.

Furthermore, we found that the child impact factor was higher than the family impact across groups but most dramatic for the conditions group. This reflects the higher distress levels of children when family accommodation is not performed. It is important to keep in mind that this distress occurs to some extent in typically developing children. For example, our sample included 10 parents who reported child impact (child impact scores between 0.25 and 0.75) but no accommodations (mean = 0). This may represent parents who are not cooperating with their child’s demands for avoidance or are not taking part in their rituals and thus, stress is higher for the child. The even higher levels of child impact scores in the conditions group may relate to the higher frequency of family accommodations, and the children’s greater difficulty in self-regulating under such conditions of negative emotionality. In this group, the rate of moderate-extreme ratings for family impact was 22–27%, while the rate of child impact items was 30–44%. Similar findings were reported in a pediatric anxiety sample (Lebowitz et al., 2013): lower levels of family distress/impact (70.7%) and higher scores for the consequences of accommodations upon the child (85.3%). This supports the common nature of family accommodations, in which lack of accommodating leads to increased child distress, regardless of the type of disorder.

This study provides a unique opportunity to examine and compare family accommodations in typical and atypical populations. Prevalence of accommodations in families raising typically developing children can serve as a baseline for assessing disability/impairment. As we expected, we found a very low prevalence of accommodation in the typical group; prevalence rating of daily and 3–6 times a week across items ranged from 3.2 to 12.2%. For the conditions group, the prevalence rating of daily and 3–6 times a week across items ranged from 4.2% (avoid places/change schedule or recreation) to 23% (enable child not to perform self-care). Family adjustments and changes in the environment and in routines are part of the normal behavior of a functioning family. Typical accommodations in this sample were providing items to reduce sensitivity and help in avoiding irritating discomfort. Among the conditions group, avoidance was much more common than changes in routines. Performing activities instead of the child was also observed more often among this group. Typical levels and types of accommodation are rarely discussed in the family accommodation literature. This study highlights the notion that family accommodation occurs to some extent in the general population and is not merely an indicator of abnormality. Further research into thresholds for impairing accommodations can enhance the clinical utility of family accommodation scales.

The higher FASENS scores reported in families with children with developmental and medical needs could be due to several reasons:

1. Increased caregiver burden: Family accommodation was previously associated with deficits in emotional regulation (Helbig-Lang et al., 2015; Reuman and Abramowitz, 2018). The burden of raising a child with a medical condition or special needs may also lead to a decrease in emotional regulation among caregivers and as a result to increased cooperation with their children’s non-adaptive behaviors.
2. Increased caregiver’s worry and protectiveness: This may occur particularly when there is inherent uncertainty in the health condition (e.g., epilepsy, Tourettes syndrome, and asthma). In some of these conditions, the family aims to avoid the child’s outburst as with respect to fear-based disorders (Reuman and Abramowitz, 2018). Parents of children with developmental and medical needs may express too much empathic concern with their child’s difficulties, and thus, cooperate with them and not expect them to self-manage these difficulties (Reuman and Abramowitz, 2018).
3. Evidence for elevated anxiety/distress and SOR in some of these conditions: The conditions group included children with conditions which often involve SOR comorbidity, for example: ADHD (Lane and Reynolds, 2019), ASD (Lane et al., 2012), allergies (Engel-Yeger et al., 2007), and general developmental delay (Rogers et al., 2003). It is expected that higher rates of SOR would lead to higher rates of accommodation. In addition, children with the developmental difficulties noted above (i.e., ADHD, ASD, allergies, etc.) present lower capacity to regulate distress (e.g., Mazefsky, 2015; Sullivan et al., 2015) and experience higher levels of distress. This is consistent with evidence showing that severity of anxiety in the child is associated with more parental accommodations (Storch et al., 2010).

Accommodation in the current sample tended to occur for younger parents of younger children, consistent with previous family accommodation evidence (Jones et al., 2015). Accommodation was also more likely to occur in families with an older sibling (potentially implying higher burden). It is noteworthy that the current sample represents larger families relative to the world^2^ ; with 2–7 children per family. In addition, the average age of children who were the eldest in our sample was about 5 years. This might explain the significant need for family accommodations, especially with several young children in the house.

### Limitations and Future Research

The conditions group was heterogeneous in terms of the child’s disability, with very different family burden and child anxiety levels. It is unclear whether families of children with health conditions report overall higher family accommodations or are responding specifically to the sensory symptoms of their children. In addition, we found no literature concerning family accommodations for children with a chronic medical condition. Further research can examine whether there are distinct sensory accommodations for specific clinical groups. There is also a need to determine family accommodations that stem from the child’s SOR versus anxiety or obsessions that develop in addition to the SOR. In this study, we did not measure the child’s or mother’s anxiety levels, which are important to characterize, to understand the mechanisms involved in the emergence of family sensory accommodations. Future research assessing potential factors contributing to family accommodation is needed. These include medical condition and special needs of family members, interventions, and other services that the family consumed, and demographic data that could influence family accommodation. Anxiety of both children and parents should be monitored considering previous findings (Kerns et al., 2017) about child and parents’ emotional dysregulation and the tendency of the family to accommodate. As our sample included only families of two-parent household, we suggest conducting a study with a larger and more representative sample that will allow to compare the effect of family structure on the tendency to accommodate SOR. The utility of the FASENS as an outcome measure requires examination of test-retest reliability.

### Clinical Implications

The FASENS adds important implications to practice by highlighting: (1) that mapping parental accommodations is critical for understanding SOR symptoms as parental behavior plays a significant role in both maintaining and exacerbating certain symptoms such as rituals, avoidance, tantrums. (2) Often children do not cooperate with interventions and the way to treat them is by including their parents in the process. The FASENS questionnaire can help parents understand their child’s difficulties and how they are retained within the family unit. (3) This tool can be used in occupational therapy applying a Family-Centered Care approach to encourage the involvement of parents in therapy.

This preliminary study examined family accommodations related to sensory sensitivity as part of the effort to study family accommodations for conditions other than anxiety and OCD (Shimshoni et al., 2019). These conditions include developmental populations like ADHD, ASD and of course, SOR. To date there are targeted interventions that address family accommodations, in cases of anxiety disorders and other psychopathologies (e.g., Peris et al., 2017; Lebowitz et al., 2020). Considering sensory family accommodation may open new opportunities for developing family oriented sensory interventions.

## Data Availability Statement

The raw data supporting the conclusions of this article will be made available by the authors, without undue reservation.

## Ethics Statement

The studies involving human participants were reviewed and approved by The Ethics Committee of the Faculty of Social Welfare and Health Sciences, University of Haifa, Israel. The patients/participants provided their written informed consent to participate in this study.

## Author Contributions

AB-S was involved in the research design and management, data collection and analysis, and manuscript writing. TP contributed to the measure design and manuscript writing. EL contributed to the measure design and manuscript preparation. All authors contributed to the article and approved the submitted version.

## Conflict of Interest

The authors declare that the research was conducted in the absence of any commercial or financial relationships that could be construed as a potential conflict of interest.

## Publisher’s Note

All claims expressed in this article are solely those of the authors and do not necessarily represent those of their affiliated organizations, or those of the publisher, the editors and the reviewers. Any product that may be evaluated in this article, or claim that may be made by its manufacturer, is not guaranteed or endorsed by the publisher.

## Footnotes

1 Ben-Sasson, A., Podoly, T. Y., and Leibowitz, E. (2020). *Family Accommodations Scale for Sensory Over-Responsivity. Unpublished manual*.

2
https://data.oecd.org/pop/fertility-rates.htm
