# Supplementary material for: Family Accommodation Scale for Sensory Over-Responsivity: A Measure Development Study
Source: Front Psychol. 2022 May 16;13:867508. doi: 10.3389/fpsyg.2022.867508 (PMC9150753; doi:10.3389/fpsyg.2022.867508)
Supplement: Supplementary file 1 [file Table_1.docx]

Supplementary Material

supplementary 1. Distribution of FASENS Items

| **Items** | Total (n=301) | | Typical | | Conditions (N=48) | |
| --- | --- | --- | --- | --- | --- | --- |
|  | N | % | N | % | N | % |
| **Assistance in sensory avoidance in the past month** | | | | | | |
| How many times have you promised your child that he/she will not be in contact with the feared sensory stimulus? | | | | | | |
| Never  1–3 times a month  1–2 times a week  3–6 times a week  Daily | 183  65  25  16  12 | 60.8  21.6  8.3  5.3  4 | 154  56  23  10  10 | 60.9  22.1  9.1  4  4 | 29  9  2  6  2 | 60.4  18.8  4.2  12.5  4.2 |
| How many times have you given your child items that help reduce his/her sensory sensitivity? | | | | | | |
| Never  1–3 times a month  1–2 times a week  3–6 times a week  Daily | 160  68  33  17  23 | 53.2  22.6  11  5.6  7.6 | 139  55  28  14  17 | 54.9  21.7  11.1  5.5  6.7 | 21  13  5  3  6 | 43.8  27.1  10.4  6.3  12.5 |
| How many times have you let your child avoid performing daily activities because of sensory sensitivity? | | | | | | |
| Never  1–3 times a month  1–2 times a week  3–6 times a week  Daily | 163  77  31  20  10 | 54.2  25.6  10.3  6.6  3.3 | 144  65  25  11  8 | 56.9  25.7  9.9  4.3  3.2 | 19  12  6  9  2 | 39.6  25  12.5  18.8  4.2 |
| How many times have you helped your child avoid stimuli or situations that cause sensory sensitivity? | | | | | | |
| Never  1–3 times a month  1–2 times a week  3–6 times a week  Daily | 137  96  33  11  24 | 45.5  31.9  11  3.7  8 | 118  82  27  6  20 | 46.6  32.4  10.7  2.4  7.9 | 19  14  6  5  4 | 39.6  29.2  12.5  10.4  8.3 |
| Did you avoid activities like going places or being with people because of your child's sensory sensitivity? | | | | | | |
| Never  1–3 times a month  1–2 times a week  3–6 times a week  Daily | 238  36  12  10  5 | 79.1  12  4  3.3  1.7 | 204  31  5  9  4 | 80.6  12.3  2  3.6  1.6 | 34  5  7  1  1 | 70.8  10.4  14.6  2.1  2.1 |
| Did you allow your child to behave oddly or inappropriately because of his sensory sensitivity? | | | | | | |
| Never  1–3 times a month  1–2 times a week  3–6 times a week  Daily | 205  55  22  9  10 | 68.1  18.3  7.3  3  3.3 | 185  44  11  8  5 | 73.1  17.4  4.3  3.2  2 | 20  11  11  1  5 | 41.7  22.9  22.9  2.1  10.4 |
| Did you do things you would not have done had it not been for your child's sensory sensitivity?**^a^** | | | | | | |
| Never  1–3 times a month  1–2 times a week  3–6 times a week  Daily | 186  71  22  13  9 | 61.8  23.6  7.3  4.3  3 | 163  61  15  9  5 | 64.4  24.1  5.9  3.6  2 | 23  10  7  4  4 | 47.9  20.8  14.6  8.3  8.3 |
| Have you changed yours or your family's schedule because of your child's sensory sensitivity? | | | | | | |
| Never  1–3 times a month  1–2 times a week  3–6 times a week  Daily | 224  48  17  4  8 | 74.4  15.9  5.6  1.3  2.7 | 192  40  11  4  6 | 75.9  15.8  4.3  1.6  2.4 | 32  8  6  0  2 | 66.7  16.7  12.5  0  4.2 |
| **Changes and modifications in family behaviors in the past month** | | | | | | |
| Did you have to do things that were naturally your child's responsibility because of the sensory sensitivity? | | | | | | |
| Never  1–3 times a month  1–2 times a week  3–6 times a week  Daily | 199  50  29  11  12 | 66.1  16.6  9.6  3.7  4 | 177  37  24  7  8 | 70  14.6  9.5  2.8  3.2 | 22  13  5  4  4 | 45.8  27.1  10.4  8.3  8.3 |
| Did you make changes in your physical home environment because of your child’s sensory sensitivity?**^a^** | | | | | | |
| Never  1–3 times a month  1–2 times a week  3–6 times a week  Daily | 232  44  14  2  9 | 77.1  14.6  4.7  0.7  3 | 196  38  11  2  6 | 77.5  15  4.3  0.8  2.4 | 36  6  3  0  3 | 75  12.5  6.3  0  6.3 |
| Have you changed your or your family's leisure or recreation habits because of your child's sensory sensitivity? | | | | | | |
| Never  1–3 times a month  1–2 times a week  3–6 times a week  Daily | 221  56  12  4  8 | 73.4  18.6  4  1.3  2.7 | 190  47  6  4  6 | 75.1  18.6  2.4  1.6  2.4 | 31  9  6  0  2 | 64.6  18.8  12.5  0  4.2 |
| Did you ask family members or other people to be considerate of your child's sensory sensitivity? | | | | | | |
| Never  1–3 times a month  1–2 times a week  3–6 times a week  Daily | 178  80  26  10  7 | 59.1  26.6  8.6  3.3  2.3 | 156  64  49  10  4 | 61.7  25.3  7.5  4  1.6 | 22  16  7  0  3 | 45.8  33.3  14.6  0  6.3 |
| **Emotional consequences to parent and family** | | | | | | |
| Did you experience distress because of the modifications and changes you made because of your child's sensory sensitivity?**^a^** | | | | | | |
| No  Mildly  Moderately  Severely  Extremely | 208  54  31  5  3 | 69.1  17.9  10.3  1.7  1 | 183  43  22  3  2 | 72.3  17  8.7  1.2  0.8 | 25  11  9  2  1 | 52.1  22.9  18.8  4.2  2.1 |
| Do the modifications and changes described above interfere with the family’s daily routine and functioning?**^a^** | | | | | | |
| No  Mildly  Moderately  Severely  Extremely | 215  55  24  6  1 | 71.4  18.3  8  2  0.3 | 193  39  15  5  1 | 76.3  15.4  5.9  2  0.4 | 22  16  9  1  0 | 45.8  33.3  18.8  2.1  0 |
| **Functional and emotional consequences to the child** | | | | | | |
| Was your child distressed when you did not help him in the ways you mentioned?**^a^** | | | | | | |
| No  Mildly  Moderately  Severely  Extremely | 167  60  51  19  4 | 55.5  19.9  16.9  6.3  1.3 | 152  48  35  14  4 | 60.1  19  13.8  5.5  1.6 | 15  12  16  5  0 | 31.3  25  33.3  10.4  0 |
| Did your child react aggressively or abusively when you did not help him/her? | | | | | | |
| No  Mildly  Moderately  Severely  Extremely | 186  62  44  8  4 | 60.8  20.6  14.6  2.7  1.3 | 161  51  31  6  4 | 63.6  20.2  12.3  2.4  1.6 | 22  11  13  2  0 | 45.8  22.9  27.1  4.2  0 |
| Did your child react angrily when you did not help him/her? | | | | | | |
| No  Mildly  Moderately  Severely  Extremely | 145  77  55  19  5 | 48.2  25.6  18.3  6.3  1.7 | 128  66  41  13  5 | 50.6  26.1  16.2  5.1  2 | 17  11  14  6  0 | 35.4  22.9  29.2  12.5  0 |
| Did your child's functioning deteriorate in situations where you did not helped him/her? | | | | | | |
| No  Mildly  Moderately  Severely  Extremely | 206  69  67  15  4 | 68.4  13  12.3  5  1.3 | 181  32  26  10  4 | 71.5  12.6  10.3  4  1.6 | 25  7  11  5  0 | 52.1  14.6  22.9  10.4  0 |

*Note.* **^a^**These items differed between conditions and typical groups based on Mann Whitney U tests.
